# Temporal dynamics of coarticulatory cues to prediction

**DOI:** 10.3389/fpsyg.2024.1446240

**Published:** 2024-09-09

**Authors:** Tugba Lulaci, Pelle Söderström, Mechtild Tronnier, Mikael Roll

**Affiliations:** ^1^Centre for Languages and Literature, Lund University, Lund, Sweden; ^2^The MARCS Institute for Brain, Behaviour and Development, Western Sydney University, Sydney, NSW, Australia

**Keywords:** speech perception, coarticulation, gating, auditory processing, prediction

## Abstract

The temporal dynamics of the perception of within-word coarticulatory cues remain a subject of ongoing debate in speech perception research. This behavioral gating study sheds light on the unfolding predictive use of anticipatory coarticulation in onset fricatives. Word onset fricatives (/f/ and /s/) were split into four gates (15, 35, 75 and 135 milliseconds). Listeners made a forced choice about the word they were listening to, based on the stimulus gates. The results showed fast predictive use of coarticulatory lip rounding during /s/ word onsets, as early as 15 ms from word onset. For /f/ onsets, coarticulatory backness and height began to be used predictively after 75 ms. These findings indicate that onset times of the occurrence and use of coarticulatory cues can be extremely fast and have a time course that differs depending on fricative type.

## 1 Introduction

Speech is a fast, continuous and transient signal that makes rapid processing necessary. It is not uncommon for words to unfold over the course of only a few hundred milliseconds (Crystal and House, 1990). Word onset fricatives can be long, reaching up to 200 milliseconds. To cope with the speed of the unfolding signal, it is advantageous for the listener to be able to gain information about the second speech sound as they are hearing the first. Optimally, this should occur already at word onset (Marslen-Wilson and Welsh, 1978). Speech sounds are influenced by their surroundings and can thus contain traces of preceding or upcoming sounds. Listeners take advantage of this fact and use *anticipatory coarticulation*—the conditioning of a speech sound on the preceding sound—to predict the upcoming phoneme (Hardcastle and Hewlett, 1999; McQueen et al., 1999). The acoustic properties of fricative portions carry coarticulatory information that can provide hints to listeners about the upcoming vowel (Soli, 1981; Yeni-Komshian and Soli, 1981). Theories propose that spoken-word recognition is influenced by articulatory features, as well as acoustic and phonetic information (Marslen-Wilson, 1987; McClelland and Elman, 1986). Based on this information, listeners can anticipate what they are going to hear based on what they have heard so far. However, the temporal profile of the perception of coarticulatory cues in word onsets has not been established. Thus, it is unknown how early in the perception of an onset fricative listeners can use coarticulatory information to predict the upcoming vowel and how this might differ among fricatives. In the present study, we used an adapted gating paradigm with short gates to investigate the early fine-grained temporal dynamics of listeners’ coarticulatory cue processing.

When perceiving speech, listeners take advantage of sub-phonemic coarticulation between words to identify the upcoming word. For example, in phrases such as *green bank*, anticipatory labialization in the first word is a cue to the upcoming /b/ in *bank*. Using eye-tracking and the visual world paradigm, Gow and McMurray (2007) showed that listeners could identify an upcoming word given anticipatory coarticulatory information around 120–160 milliseconds after the following word onset. Salverda et al. (2014) reported that listeners could anticipate an upcoming word onset using the cues from a preceding word offset, showing fixations starting from 130–170 ms after hearing the onset of a target word.

Investigations focusing on within-word cues with cross-spliced stimuli further support a facilitatory effect of coarticulation in speech perception. Beddor et al. (2013) reported that listeners use vowel nasalization introduced by an upcoming consonant as a cue in stimuli such as *bent*. In another study—with cross-spliced stimuli where stimuli were spliced as word onset from word_1_ and merged with word offset from word_2_ such as [ne]ck + ne[t]—it was found that listeners used coarticulatory information and fixated the picture of the target stimuli (e.g., *neck*) 200 ms after hearing the CV onset (Dahan et al., 2001). Their result suggested that mismatched word onsets carried enough information for listeners to identify the word offset.

Despite various studies differing in their temporal findings, studies investigating spoken word recognition have suggested that phoneme information from multiple sources can help listeners recognize the word efficiently. Behavioral studies investigating both word and non-word stimuli have shown evidence for listeners’ sensitivity to fine-grained acoustic cues. McQueen et al. (1999) observed no differences between non-word+word cross-spliced and non-word+non-word cross-spliced stimuli. This suggests that lexical status may not play an important role in early acoustic perception, but that listeners are particularly sensitive to fine-grained phonetic information. Mismatching information in formant transitions, place of articulation and release bursts can disrupt perceptual decisions, and listener sensitivity to coarticulatory cues may thus depend on the type of speech sound encountered. Marslen-Wilson and Warren (1994) tested three different articulation types—unvoiced fricatives, unvoiced plosives and voiced plosives—in CVC words and non-words mismatching in coarticulation. Their findings suggested that acoustic information in the vowel can provide misleading cues to the place of articulation of a following consonant, but those different consonants affected the perception differently: for example, unvoiced fricatives showed no significant effect in cross-spliced conditions— neither for word_1_ + word_1_ nor word_2_ + word_1_ stimuli—while voiced plosives showed a significant effect on perception, as measured by reaction times.

Gating studies of coarticulation, investigating progressively longer portions—or *gates*—of fricative sounds, have been used to probe listeners’ identification of an upcoming speech sound ahead of time, based on coarticulatory information (Kingston et al., 2016). Features of vowel height and backness have been shown to function as cues to a subset of English vowels (McMurray and Jongman, 2016). Some have proposed that listeners wait to decide until all relevant cues from the vowel onset are available (Galle et al., 2019) while other studies using gating paradigms suggest an incremental process (Kingston et al., 2016).

Prior studies have thus provided valuable information about the effect of fine-grained phonetic information on speech perception, employing various tasks and stimuli. However, less is known about the temporal dynamics and predictive use of acoustic cues in word onset coarticulation within words, particularly in unaltered stimuli that reflect natural mandibular, lingual and labial movements. The present study investigated when acoustic cues to different features of coarticulation in word onset fricatives become available to the listener.

We used an adapted gating paradigm (Grosjean, 1980) with short gates to assess the earliest time point at which listeners can use coarticulatory cues such as roundedness and tongue height and backness to predict upcoming speech sounds. Previous studies researching gradient effects of acoustic details have usually utilized a small number of cardinal vowels and various consonant combinations. In the present study, we contrasted all twenty long and short vowel allophones available in Central Swedish to explore the effects of fine-grained acoustic details. We used articulatory movements to observe the differences on the auditory signal and its effect on perception. Articulatory movements occur as a result of natural speech production. As the vocal tract changes from one state to another, following or preceding movements influence the current gesture, and thus, overlapping articulator movements are inevitable (Kent and Minifie, 1977). Articulatory movements are inherently fast and transitions to upcoming movements require pre-planned mandibular, lingual, and labial activity. Due to their complex nature, these changes are gradual and not fully understood. In the present study, we aimed to discover the perceptual effects of within-word acoustic changes based on these articulatory movements.

Real words with /s/ and /f/ onset were used in this study, motivated by those fricatives’ distinct articulatory and acoustic properties, useful in understanding the temporal dynamics of speech perception. Limiting the word onsets to two fricatives allowed us to keep the study design focused on how different vowels interact with the preceding fricatives. Only unvoiced fricatives were selected to limit the number of acoustic cues, increasing the detection of possible vowel effects in the consonant. Unlike voiced fricatives, unvoiced fricatives are produced without effective vocal fold vibration, allowing us to avoid shadowing cues due to consonant voicing. Unvoiced fricative noise duration is also known to be more extensive than that of voiced fricatives enhancing the measuring of the temporal dynamics of coarticulation (Jongman et al., 2000). The sibilant /s/ is an unvoiced alveolar fricative produced with a narrow constriction at the alveolar ridge while /f/ is an unvoiced labiodental fricative, occurring as a result of a constriction formed by upper teeth and lower lip in the frontal part of the oral cavity (Ladefoged and Johnson, 2011). The /s/ articulation lacks lip rounding in isolation form, while the unvoiced labiodental fricative /f/ involves lip articulation even in isolation. This could potentially affect the temporal dynamics and coarticulatory cues in a CV context. Indeed, previous production studies have shown stronger labial activity for /s/ when followed by a rounded vowel compared to other fricatives (Lubker and Gay, 1982; Perkell and Matthies, 1992). These articulatory and acoustic differences lead to different spectral characteristics, which could influence how coarticulatory information is perceived and used over time and could lead to different time course patterns. Specifically, alveolar fricatives are defined by high spectral energy with major peaks while labiodental fricatives show a flat spectrum (Jongman et al., 2000).

Focusing on /s/ and /f/, this study explored how these fricatives with their contrasting features affect the temporal dynamics and use of coarticulatory cues in speech perception to understand how listeners process the complex auditory signal during real-time speech perception.

## 2 Materials and method

### 2.1 Stimuli

A Central Swedish speaker recorded isolated monosyllabic words in citation form. Twenty words began with /s/ and twenty with /f/. The stimuli were recorded in a soundproof room using a U47 FET microphone positioned at a fixed distance from the speaker to maintain a consistent sound pressure level during the recordings. The auditory signal from the microphone was routed through a Universal Audio 6176 preamplifier and a RME Fireface UCX II sound interface. The recordings were made at 44.1 kHz sample rate using the Cubase 8 audio recording software.

We recorded only one speaker to minimize inter-speaker variation (Soli, 1981), focusing on listeners’ perception of different fricatives and coarticulatory patterns rather than exploring individual differences across speakers. The lists contained all Swedish long and short vowel allophones, derived from phonological representations in Riad (2014), presented in Table 1. The /f/ and /s/ stimulus lists thus shared the same vowels (for the stimulus list along with word frequency and lexical competition statistics, see Supplementary Table S1). Each word was recorded 10 times, creating 400 unique stimuli in total. A Praat script identified word onsets based on a predefined intensity threshold (>35 dB).

**Table 1:** Swedish long and short vowel allophones used in the stimuli adapted from Riad (2014).

	Front	Central	Back
High	**[ʏ]** [i:] **[y:]** [ɪ]	**[ʉː]**	**[uː]**
Mid	**[øː]** [e:] **[ø̞]** [ɛ̝] [ɛː]	**[ɵ]** **[œ]**	**[oː]** **[ɔ]**
Low	**[œː]** [æ] [æ:]	[a]	[ɑː]

Rounded vowels are in bold.

The vowels were grouped based on the articulatory features height, backness, and roundness according to Riad (2014).

Word-onset fricative proportions within the individual word were calculated to test a possible effect of duration on listeners’ accuracy levels (/f/ onset *M =* 23.36, *SD =* 1.98; /s/ onset *M =* 25.30, *SD* = 2.06). A two-sample t-test was performed to compare fricative proportions between /s/ and /f/ onset words, revealing a statistically significant difference between word onset fricatives (*t* = −3.0214, *df* = 38, *p* = 0.004). Duration differences both in word onset (*t* = −5.458, *df* = 38, *p* < 0.001) and the whole word (*t* = 0.29491, *df* = 38, *p* = 0.769) were calculated additionally for further analysis. Word onset fricative duration and proportion of fricative durations were thus different for /f/ and /s/, while word duration did not differ significantly between the two groups. A further analysis was carried out to control any possible influences of fricative duration and accuracy but this had no effect on response accuracy (/f/ word onsets, *p* = 0.463, /s/ word onsets, *p* = 0.076).

The gating paradigm (Grosjean, 1980) was used to investigate speech perception in real-time with a focus on coarticulatory cues. This paradigm provides the listener with incrementally longer portions of the speech signal called *gates*, the procedure makes it possible to detect when listeners process different cues in the speech.

A Praat script was used to divide words into four gates starting from word onset. The first gate was 15 ms, the second gate was 35 ms (adding 20 ms to preceding gate), and the third was 75 ms (adding 40 ms to preceding gate). The final gate was 135 ms (adding 60 ms to preceding gate). The longest gate—135 ms from word onset—was shorter than the full duration of the onset fricatives of all words. Therefore, the gates only contained word onset fricatives. The design allowed us to investigate at which point of the unfolding speech signal the listeners had received enough information to accurately identify the word, and, thus, the vowel.

In addition to word and onset duration, any potential effect of word frequency on response accuracy was controlled, using frequency statistics from the Swedish language corpus PAROLE (Borin et al., 2012).

#### 2.1.1 Acoustic stimulus analysis

Considering the natural, unaltered state of the stimuli, we analyzed the auditory signal to measure possible changes in the basic spectral properties of the fricatives due to the upcoming speech sounds. To this end, we calculated their Center of Gravity (CoG) using Praat (Boersma and Weenink, 2021). A Praat script was used to complete the CoG analysis. The script first converted the 15 ms, 35 ms, 75 ms, and 135 ms sound files to spectrum using the built-in Praat function “to spectrum” fast Fourier transform (FFT). Following this, the Praat function “get centre of gravity” calculated the spectral center of gravity for each gate. The center of gravity function calculates the average frequency weighted by the power spectrum for each gate (Equation 1). This approach provided consistency across the analyses by applying standardized parameters for CoG calculation.

(1) $$\mathrm{CoG}=\frac{\int_{0}^{\infty }f{\left|S\left(f\right)\right|}^{2}df}{\int_{0}^{\infty }{\left|S\left(f\right)\right|}^{2}df}$$

for the spectrum given by S(*f*), where *f* is the frequency (Boersma and Weenink, 2021).

**Figure 1:**
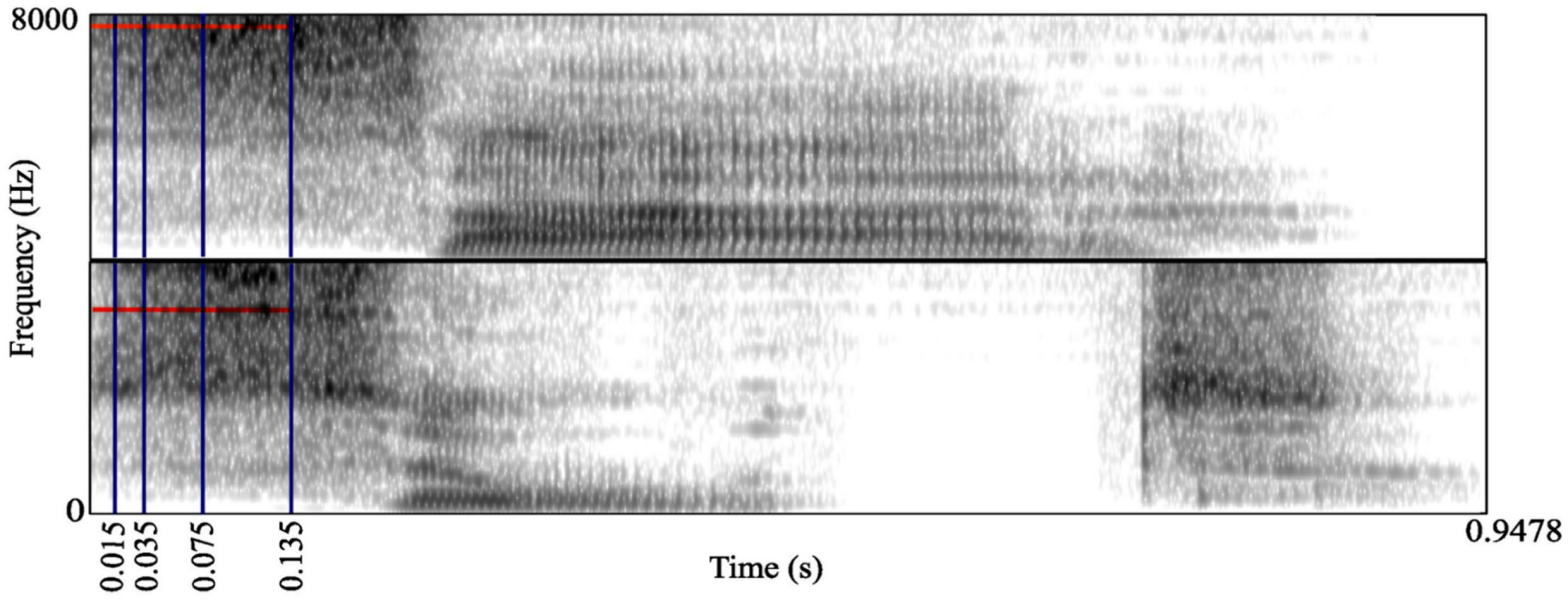
Gates (in dark blue) and spectral center of gravity (CoG) (in red) marked in the spectrograms of [sæːr] (top), CoG = 7,619 Hz, and [suːt] (bottom), CoG = 6,401 Hz.

We employed a similar method to that used by Lulaci et al. (2022) and Wikse Barrow et al. (2022), who demonstrated that spectral CoG varied between fricatives, and that different places of articulation also affected the CoG. The difference in spectral energy for the same word onset /s/ but with different following vowel in Figure 1 can be observed in the spectrogram.

The dataset consisted of 20 /s/ and 20 /f/ words. Each word was recorded 10 times resulting in 200 unique sound files for /f/ and 200 sound files for /s/. These 200 sound files were further divided into four gates, yielding 800 sound files in total for /s/ and 800 sound files in total for /f/. The resulting 1,600 unique gates (200 sound files for each gate) were included in the CoG measurement. Each model consisted of all the 200 unique sound files per gate to maintain a diverse sample and to capture the variability in the acoustic properties of the stimuli. Statistical analyses were performed using R Core Team (2022) with the lme4 package (Bates et al., 2015). Generalized linear models were used to test the relation between CoG values and vowel features. We used 4 different models to assess each gate separately for each fricative. Since we grouped the vowels used based on their articulatory features to assess the possible lingual and labial effects on the auditory signal, CoG was the dependent variable, while roundedness, height, and backness were independent variables. As shown in Table 2 /s/, roundedness and backness affected CoG in all gates (all *p* < 0.05), while height had no effect. The picture was slightly more complex for /f/ fricatives. In the 15-ms gate, roundedness was related to CoG (*p* = 0.012), while backness and height were not. There were no significant effects on CoG in the 35-ms and 75-ms gates, but the 135-ms gate yielded effects of height, backness, and roundedness (all *p* < 0.001).

**Table 2:** Lingual and labial movement effects on CoG.

	/s/					/f/				
		EST	SE	*t*	*p*-value		EST	SE	*t*	*p*-value
15 ms	Height	−131.81	92.88	−1.419	0.157	Height	14.77	184.90	0.080	0.936
	Backness	−235.97	89.02	−2.651	**0.008**	Backness	−69.46	177.23	−0.392	0.695
	Roundedness	−1120.92	149.10	−7.518	**< 0.001**	Roundedness	−752.24	296.83	−2.534	**0.012**
35 ms	Height	11.15	52.21	0.213	0.831	Height	−1.268	133.528	−0.009	0.992
	Backness	−265.59	50.04	−5.307	**< 0.001**	Backness	−26.760	127.988	−0.209	0.835
	Roundedness	−1038.26	83.81	−12.389	**< 0.001**	Roundedness	−323.743	214.358	−1.510	0.133
75 ms	Height	12.92	49.00	0.264	0.792	Height	−86.07	133.94	−0.643	0.521
	Backness	−277.06	46.97	−5.899	**< 0.001**	Backness	−155.23	128.38	−1.209	0.228
	Roundedness	−998.10	78.67	−12.688	**< 0.001**	Roundedness	36.90	215.02	0.172	0.864
135 ms	Height	−20.61	53.70	−0.384	0.702	Height	−196.89	18.22	−10.806	**< 0.001**
	Backness	101.52	51.47	1.972	**0.050**	Backness	97.69	17.46	5.594	**< 0.001**
	Roundedness	−502.58	86.21	−5.830	**< 0.001**	Roundedness	−145.96	29.25	−4.990	**< 0.001**

Statistically significant *p*-values are shown in bold.

Results indicated that labial and lingual movements due to the upcoming vowel influenced CoG both for the preceding /s/ and /f/, but the effect of CoG was less apparent in /f/ compared to /s/.

### 2.2 Participants

Twenty native speakers of Central Swedish (11 female, mean age = 24.6 years, SD = 3.7, range = 20–33 years) participated in the study. All participants had normal hearing (< 20 dB) as assessed by pure-tone audiometry (Callisto audiometer, Interacoustics, with Radioear DD450 headphones), and all participants had normal or corrected-to-normal eyesight. A language background questionnaire showed that none of the participants was early bilingual.

### 2.3 Procedure

Participants were given information sheets containing a detailed description of the study and signed the consent forms. Participants then filled out questionnaires about their language background and demographic information. They were seated in a comfortable position 50–60 centimeters away from a 15.6-inch (1600×900 resolution) computer screen in a sound-isolated room. Their eye level was aligned with the center of the screen, where the stimuli were presented. A standard keyboard was used to record their responses.

The experiment was conducted using PsychoPy (Peirce et al., 2019). It began with an information screen to provide details about the task and instructions. Three familiarization trials were included to prepare the participants for the task. The audio stimuli were presented binaurally via headphones (Philips Fidelio X3). The sound level was adjusted to a comfortable listening level during the first familiarization trial and remained the same until the end of the test.

For the experiment, a two-alternative forced choice paradigm was used. That is, for each trial, the participants were instructed to listen to the audio stimuli (words) and select one of the two words (e.g., ***sal*** *+ sått* or ***sal*** *+ sot*, etc.) simultaneously displayed on the screen that they thought they were hearing, as fast as possible using arrow keys (right/left).

Participants were presented with 760 trial groups (380 for /s/ and 380 for /f/) in randomized order. Each trial group contained four gates where participants listened to isolated stimuli presented in 15 ms, 35 ms, 75 ms, and 135 ms, respectively, for a total of 3,040 tokens.

Trials within each trial groups were presented in fixed order. That is, participants were initially presented with the 15-ms gate. After listening to the first gate, they were asked to choose between two words presented on the screen. Following their response to the first gate, the experiment moved into the second gate and participants were presented with the 35-ms gate to make a second decision about the same word they had listened to, this time starting from word onset until 35 ms. Again, after their selection, the experiment proceeded to the 75-ms gate and after they had listened to the 75-ms gate and made their decision, the experiment moved on to the last gate (135 ms). After the participants had listened to the 135-ms gate and chosen the word on the right or left using arrow keys, the experiment moved on to the last step. Finally, the full word was played without gating and without asking the participant to identify the word. This design allowed participants to hear the full word and have the chance to know whether they had identified the word correctly or not.

Words were only compared within their respective onset groups (e.g., words in the /s/ onset list were compared with other words in /s/ onset list, and similarly for /f/ onset list words). A key point in the study design was that no word was compared with itself. Therefore, within each list of 20 words, every word was compared with the other 19 words. This resulted in a total of 380 comparisons for /s/ and 380 comparisons for /f/ (19 comparisons of 20 words). Each trial contained four gates. This structure generated a total of 1,520 gates for /s/ and 1,520 gates for /f/ per participant. In total, 3,040 gate decisions were completed per participant (380 word pairs × 4 gates for /s/ + 380 word pairs × 4 gates for /f/).

### 2.4 Statistical analysis

All behavioral analyses focused on listeners’ accuracy in determining the unfolding word, with the aim of examining the earliest point where coarticulation-based information became available to the listeners, as well as exploring the differences in predictability between distinct features of the upcoming vowel present in the onset fricative. Four respective statistical models assessed the relation for the 15, 35, 75, and 135 ms gates. Each participant completed 760 trials, each consisting of 4 gates with 15 ms, 35 ms, 75 ms and 135 ms gates. Therefore, 3,040 observations per participant (760 trials × 4 gates) were observed. Each gate had 380 observations per participant, and when data from all 20 participants were rendered together, a total of 7,600 observations per gate were used in the statistical analysis.

We used generalized linear mixed models (GLMM) to assess the effects of the predictor variables on the response accuracy (the dependent variable). The model used a logit link function and included random intercepts of participants and items. Fixed and random effects were estimated using maximum likelihood with Laplace approximation. Fixed effects were articulatory vowel features: height, backness and lip roundedness. In addition, word frequency was added as a fixed effect to ensure that the observed articulatory effects on accuracy were not influenced by word frequency. The levels of the fixed effects were based on the articulatory difference between the vowel following the onset fricative. Roundedness had two levels: same (no difference in roundedness) and Different (difference in roundedness). Three levels of backness were: Same (no difference in backness), Different (front vs. central and central vs. back), and Very Different (front vs. back). Height also had three levels: Same (no difference in height), Different (low vs. mid and mid vs. high), and Very Different (low vs. high). Since we compared three different articulatory features and word frequency in the same dataset, Bonferroni correction was applied and the alpha level was set to 0.0125 (0.05/4). Separate models were used to rule out effects of fricative duration on response accuracy. A linear model was used to assess a possible relation between average response accuracy percentage and fricative duration averages.

## 3 Results

### 3.1 Accuracy of /s/ and /f/

Articulatory movements affected both temporal perception patterns and accuracy. The results of the linear mixed models are presented in Table 3.

**Table 3:** Statistical analysis results of articulatory movements of /s/ and /f/ onset words and word frequency effects on auditory perception.

/s/					/f/				
	EST	SE	*z*	*p*-value		EST	SE	*z*	*p*-value
**15 ms**					**15 ms**				
Height	−0.05290	0.04087	−1.294	0.196	Height	0.06668	0.03730	1.788	0.073
Backness	0.01350	0.03203	0.421	0.673	Backness	0.02520	0.03008	0.838	0.402
Roundedness	0.21499	0.04763	4.514	**< 0.001**	Roundedness	0.06097	0.04669	1.306	0.191
Word frequency	−0.02149	0.03111	−0.691	0.490	Word frequency	−0.02476	0.02708	−0.914	0.360
**35 ms**					**35 ms**				
Height	0.01839	0.04295	0.428	0.669	Height	0.05889	0.03907	1.507	0.132
Backness	0.04406	0.03341	1.319	0.187	Backness	0.03383	0.03103	1.091	0.275
Roundedness	0.58771	0.04910	11.969	**< 0.001**	Roundedness	0.06618	0.04701	1.408	0.159
Word frequency	0.01204	0.03325	0.362	0.717	Word frequency	−0.02859	0.02909	−0.983	0.326
**75 ms**					**75 ms**				
Height	0.02809	0.04506	0.623	0.533	Height	0.16620	0.04243	3.917	**< 0.001**
Backness	0.04403	0.03484	1.264	0.206	Backness	0.08217	0.03273	2.510	**0.012**
Roundedness	0.94795	0.05164	18.358	**< 0.001**	Roundedness	0.05191	0.04771	1.088	0.276
Word frequency	−0.06445	0.03391	−1.901	0.057	Word frequency	−0.04008	0.03263	−1.228	0.219
**135 ms**					**135 ms**				
Height	0.01796	0.07084	0.254	0.800	Height	0.11397	0.06644	1.715	0.086
Backness	0.01489	0.05293	0.281	0.778	Backness	0.02979	0.04984	0.598	0.550
Roundedness	0.79236	0.07503	10.560	**< 0.001**	Roundedness	0.03021	0.06993	0.432	0.655
Word frequency	−0.06289	0.05804	−1.084	0.279	Word frequency	−0.04840	0.05523	−0.876	0.380

Statistically significant *p*-values are shown in bold.

Roundedness in the fricative /s/ significantly increased the accuracy in the 15-ms gate and remained significant across all the gates (*p* < 0.001), while backness, height, and word frequency did not influence the results.

/f/ onset words showed a different pattern. Backness enhanced the accuracy in the 75-ms gate (*p* = 0.012). Difference in height also increased the accuracy in the 75-ms gate (*p* < 0.001). The 15-, 35- and 135-ms gates did not show any effect of height, roundedness, or backness on accuracy. Word frequency did not have any significant effect in any gate.

**Figure 2:**
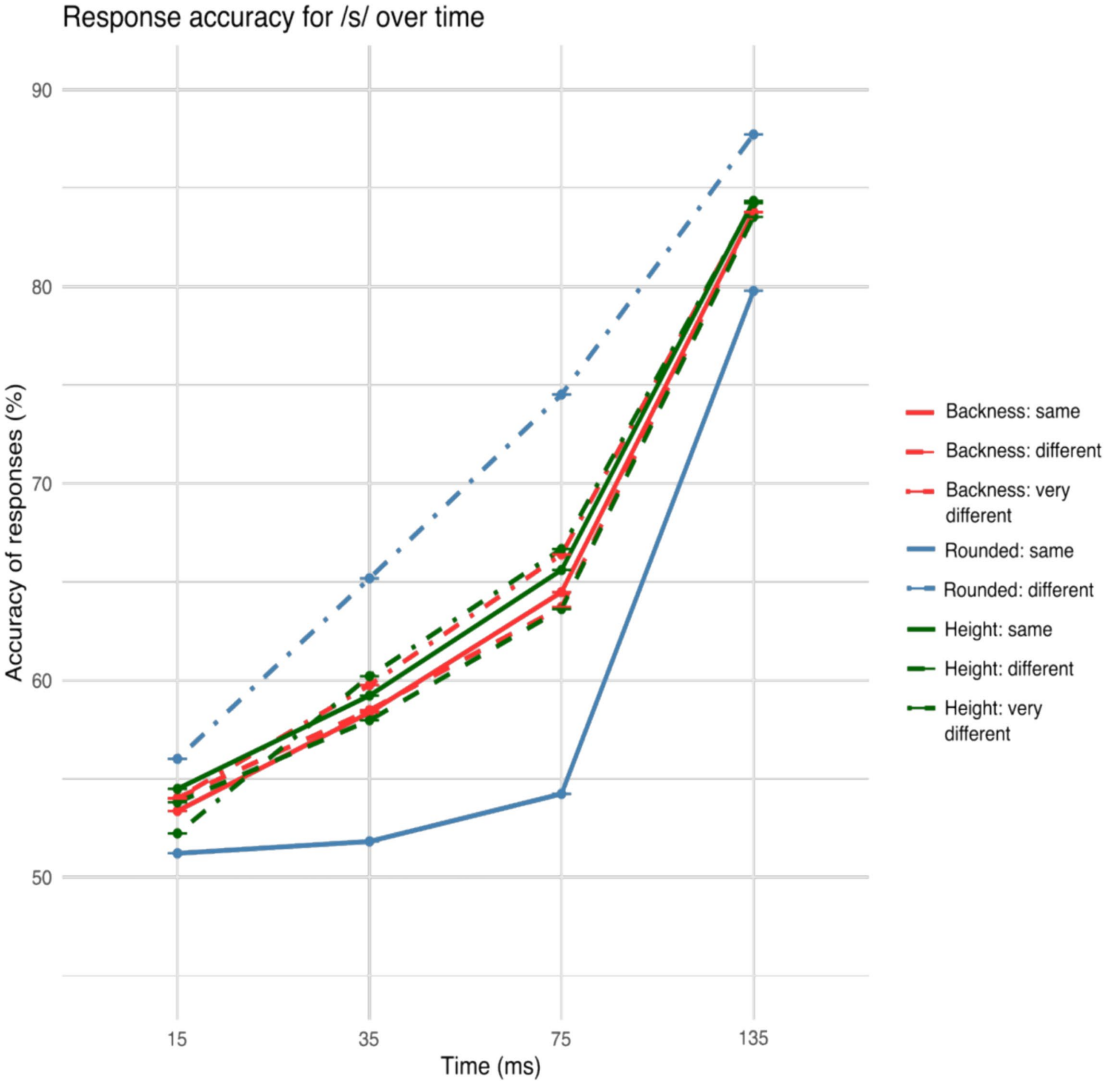
Accuracy of the responses across the /s/ onset fricative gates for particular articulatory movements. A clear effect of roundedness difference between the visually presented target words on auditory vowel identification is seen already in the 15 ms gate and throughout the onset fricative.

**Figure 3:**
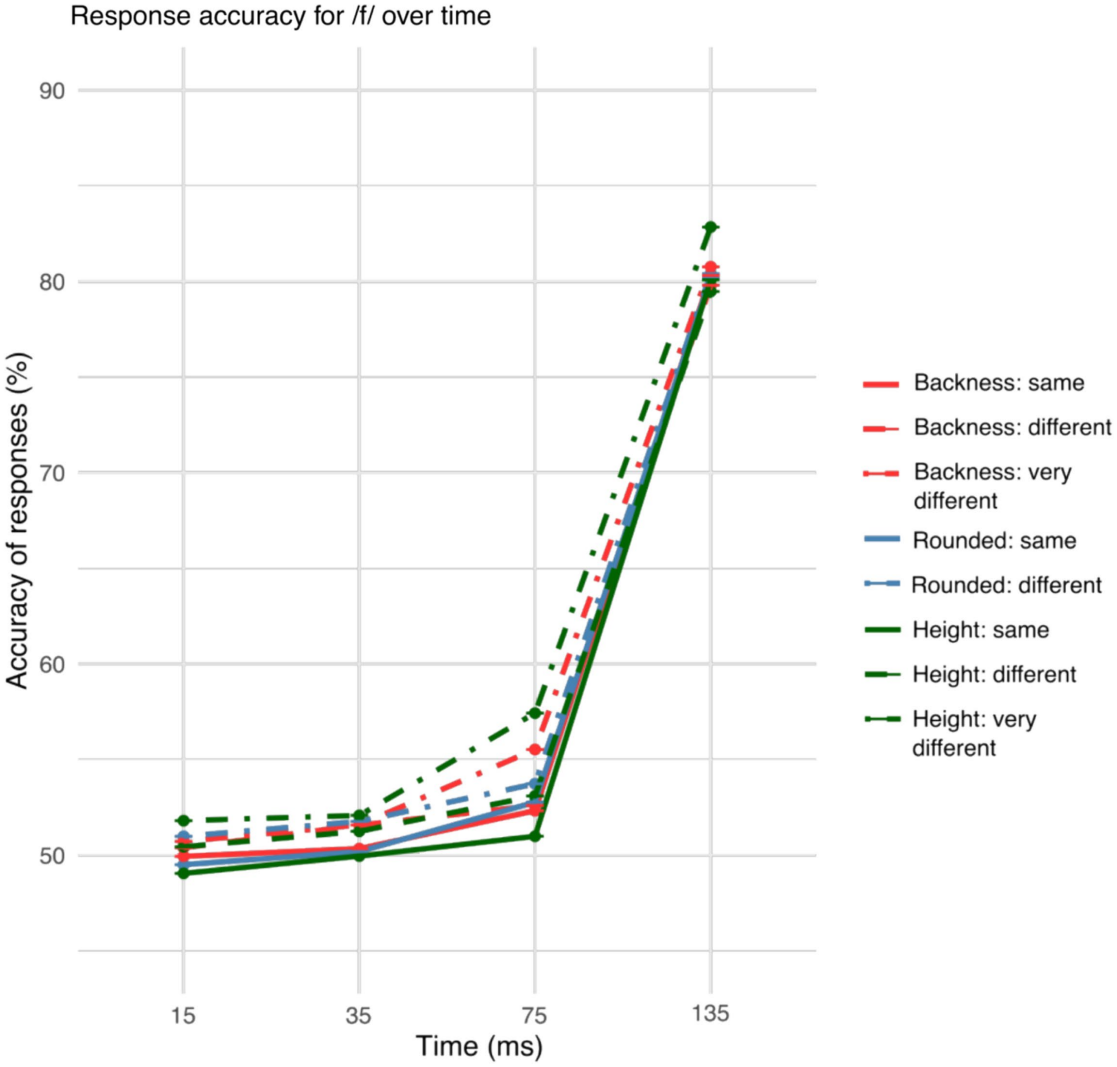
Accuracy of the responses across the gates of /f/ for differences in articulatory features. Height and backness differences between the visual target words improved vowel identification in the 75-ms gate of the auditorily presented onset fricative.

The accuracy showed a general increase over gates both for /s/ and /f/ onset words as can be seen in Figures 2, 3.

Figures 2, 3 illustrate the different accuracy patterns based on coarticulation of /s/ and /f/ onset words.

## 4 Discussion

We traced the temporal dynamics of coarticulatory changes in onset fricatives, and their predictive utility and processing, using a behavioral gating paradigm. The acoustic analysis of the stimuli indicated that the auditory signal within the onset fricatives changed due to articulatory patterns, with labial movements affecting the signal the most: for the onset sibilant fricative /s/, acoustic changes due to an upcoming rounded vowel were available to the listener as early as in the first 15 milliseconds of the fricative. Both the acoustic and behavioral effects of roundedness increased over time until 135 milliseconds, such that response accuracy increased over time, along with the effect of center of gravity (CoG) frequency, which indicates that accuracy increased as the acoustic information in the fricative became more predictively informative (see Tables 2, 3).

The labiodental fricative /f/ showed a different timeline compared to /s/ both in acoustic analysis and behavioral results. Roundedness was a significant predictor for the acoustic cues of coarticulation during the first 15 milliseconds. Acoustic correlates of height and backness were present in the later parts of the fricative (135 milliseconds). Behavoiral results showed rounded vowels had no effect on response accuracy words with /f/ onset, but listeners could use vowel height and backness to predict the upcoming vowel later in the fricative, from 75 ms onwards. Thus, labial movements were less important to listeners than lingual movements in providing helpful cues in perceiving the unfolding /f/ fricative-onset word. Acoustic correlates suggested a strong relation between labial movement and CoG for words with /s/ and /f/ onsets as early as 15 ms into fricative onsets. However, CoG could not capture lingual movements until 135 ms after vowel onset.

The results provide insights into the effects of coarticulation evolving over time. The fine-grained coarticulatory information derived from different CV sequences varied and was available for the different fricatives at varying time points. Sibilants like /s/ and non-sibilants like /f/ have different patterns in terms of carrying acoustic cues and differences perceived by listeners. Soli (1981) tested coarticulation in production in CV contexts with sibilant fricatives starting from 10 ms before fricative offset and five preceding LPC spectra, showing that coarticulatory cues were present at least 30–60 ms before vowel onset. While our /s/ results align with Soli (1981) and Yeni-Komshian and Soli (1981) in terms of backness and roundedness, both the acoustic and perception data suggest an earlier time course of perceptually useful acoustic coarticulatory information in the onset fricative in the present study.

Spectral shape is a product of how the sound is produced. The size and shape of the oral cavity and constriction or narrowing affect the signal. The lack of well-defined spectral shapes in labiodental fricatives creates evenly distributed/flat spectra without visible peaks at specific frequencies. Thus, while sibilants produce distinguishable spectral moments, non-sibilants do not (Jongman et al., 2000; Shadle et al., 2023). This might explain why words with /f/ onsets demonstrated delayed temporal profiles compared to /s/ onset words in the present study, highlighting the important role of fine-grained auditory information in human perception.

A number of previous studies have investigated CV contexts concentrating on a variety of fricatives and provided valuable insights (Jongman et al., 2000; Nittrouer et al., 1989; Soli, 1981; Yeni-Komshian and Soli, 1981). In the present study, we investigated fine-grained differences between various CV contexts using only 2 fricatives but 20 vowels in Central Swedish. With this approach, we observed effects in a diverse set of stimuli, and we managed to capture the subtle changes over time of the entire fricative onset in 20 different coarticulatory contexts and detected correlations between features present in the fricative onset and features of the vowel.

In the behavioral results for /s/, height and backness were insufficient to provide cues during the very early gates, in contrast to roundedness. Speech production studies on anticipatory coarticulation in English have shown stronger labial activity for /s/ than other consonants in American English (Lubker and Gay, 1982; Perkell and Matthies, 1992). Lubker and Gay (1982) observed that coarticulatory cues in production might be language-specific: native Swedish speakers show stronger labial protrusion in rounded vowel production compared to native American English speakers. This suggests that labial coarticulation may be a learned behavior. Among other articulatory features, lip rounding has also been reported as a stronger indicator of a feedforward process of articulation (Bell-Berti and Harris, 1979). Furthermore, it has been shown that the anticipatory effect of rounded vowels is present across multiple preceding sounds, not only just before the vowel, which indicates an important effect of lip rounding in perception (Öhman, 1966). Our results show that listeners can use the distinct and robust information from the labial gesture to anticipate the nature of the vowel as early as word onset (15 milliseconds into the initial fricative). These results point to lip rounding as a strong gesture, in both production and perception. The auditory signal was influenced differently depending on articulatory movements. While /s/ was generally affected by roundness, other movements failed to cause a major change in the signal (or the effect of roundness may have overshadowed other effects). Coarticulatory acoustic correlates and behavioral accuracy results varied as a function of the consonant-vowel context. This highlights the important influence of coarticulation, both in perception and in the acoustics of the speech signal.

Considering the rapid nature of the speech signal, humans appear to predict relevant information about what they are about to hear to help them cope with ambiguity (Bar, 2007). The brain constantly creates predictions, and in speech perception, many factors are thought to impact predictions (Marslen-Wilson, 1987; McClelland and Elman, 1986; Norris, 1994). Various factors including word frequency and duration are involved in lexical activation alongside the acoustic information. However, as shown in the present study, fine-grained phonetic features play a particularly important role in spoken-word recognition (McMurray et al., 2008; McQueen et al., 1999); lingual and labial articulatory movements due to vowel features functioned as informative cues, while word frequency did not show any effect on response accuracy.

Furthermore, coarticulatory acoustic-phonetic cues showed different temporal dynamics depending on the fricative. Words with /s/ and /f/ onsets demonstrated different spectro-temporal patterns. In particular, words with /f/ onsets had a delayed effect on the predictive use of coarticulation (75 ms) as compared to words with /s/ onsets (15 ms). The different lingual movements also yielded different patterns: height was a stronger predictor compared to backness for /f/. In several languages, backness and roundedness are related to each other. Generally, front vowels are unrounded, while back vowels tend to be rounded (Ladefoged and Maddieson, 1996). For the /f/ 75-millisecond gate of the behavioral experiment, height was a stronger predictor than backness. The labiodental feature of /f/ might affect the access to possible cues from labial movement/roundedness and reduce the effect of backness.

## 5 Conclusion

We found behavioral effects of fricative-vowel coarticulation at fricative onset (during the first 15 milliseconds), and throughout the consonant, the auditory signal affected the temporal perception patterns. Coarticulatory cues of roundedness were strong for /s/ from word onset throughout the fricative, whereas backness and height influenced the acoustics and processing of words beginning with /f/ only in the later parts (75 and 135 milliseconds) of the fricative. Since the lips are not contrastively involved, coarticulatory rounding is more free to influence the pronunciation of /s/ than that of labiodental /f/.

## Data Availability

The datasets presented in this study can be found in online repositories. The names of the repository/repositories and accession number(s) can be found at: https://osf.io/2mqu3/?view_only=b47d87fcb29045c1b1ba1e30ac8fc4d7.
