# Supplementary material for: Temporal dynamics of coarticulatory cues to prediction
Source: Front Psychol. 2024 Sep 9;15:1446240. doi: 10.3389/fpsyg.2024.1446240 (PMC11416931; doi:10.3389/fpsyg.2024.1446240)
Supplement: Supplementary file 1 [file Data_Sheet_1.docx]

Supplementary Material

Temporal dynamics of coarticulatory cues to prediction

# Supplementary Figures and Tables

|  | Stimuli | Word frequency | Lexical Competitors |  | Stimuli | Word frequency | Lexical Competitors |
| --- | --- | --- | --- | --- | --- | --- | --- |
| S/ɑ | sal | 129 | 204 | **F/ɑ** | fas | 180 | 184 |
| S/ɔ | sått | 26 | 480 | **F/ɔ** | fåll | 3 | 714 |
| S/u | sot | 49 | 265 | **F/u** | fot | 606 | 271 |
| S/o: | sås | 206 | 159 | **F/o** | fås | 88 | 144 |
| S/ɵ | suck | 243 | 235 | **F/ɵ** | full | 2381 | 259 |
| S/œ | sörjs | 3 | 201 | **F/œ** | förr | 1955 | 3065 |
| S/œ: | sir | 292 | 100 | **F/œ** | för | 248832 | 910 |
| S/ø | sök | 15 | 100 | **F/ø** | föl | 17 | 910 |
| S/ø̞ | sött | 76 | 201 | **F/ø̞** | föll | 2379 | 3065 |
| S/ʏ | sytt | 70 | 436 | **F/ʏ** | fyll | 19 | 81 |
| S/ʉ | sus | 55 | 145 | **F/ʉ** | ful | 174 | 38 |
| S/yː | syr | 82 | 224 | **F/yː** | fyr | 72 | 116 |
| S/i | sil | 13 | 118 | **F/i** | fil | 325 | 151 |
| S/ɪ | sill | 122 | 670 | **F/ɪ** | filt | 117 | 898 |
| S/eː | ses | 1408 | 465 | **F/eː** | fel | 4792 | 136 |
| S/a | sats | 176 | 1158 | **F/a** | falk | 4 | 845 |
| S/æ | särk | 5 | 472 | **F/æ** | färsk | 409 | 538 |
| S/æː | sär | 24 | 250 | **F/æː** | färd | 355 | 88 |
| S/ɛ̝ | sätt | 12587 | 472 | **F/ɛ̝** | fält | 370 | 538 |
| S/ɛː | säl | 31 | 250 | **F/ɛː** | fän | 2 | 88 |

**Supplementary Table 1.** Gating experiment stimulus word list and word frequency statistics. The lexical competitor measure is based on the onset fricative and vowel sequence

| /s/ | | | | /f/ | | | |
| --- | --- | --- | --- | --- | --- | --- | --- |
|  | **Same** | **Different** | **Very different** |  | **Same** | **Different** | **Very different** |
|  | M  (%) | M  (%) | M  (%) |  | M  (%) | M  (%) | M  (%) |
| 15 ms |  |  |  | **15 ms** |  |  |  |
| Height | 54.48 | 53.80 | 52.22 | Height | 49.05 | 50.44 | 51.80 |
| Backness | 53.36 | 54.01 | 53.99 | Backness | 49.94 | 50.39 | 50.72 |
| Roundedness | 51.20 | 56.01 | N/A | Roundedness | 49.50 | 50.98 | N/A |
| 35 ms |  |  |  | **35 ms** |  |  |  |
| Height | 59.22 | 57.96 | 60.20 | Height | 49.95 | 51.25 | 52.08 |
| Backness | 58.36 | 58.48 | 59.75 | Backness | 50.34 | 51.56 | 51.58 |
| Roundedness | 51.81 | 65.17 | N/A | Roundedness | 50.19 | 51.76 | N/A |
| 75 ms |  |  |  | **75 ms** |  |  |  |
| Height | 65.60 | 63.61 | 66.66 | Height | 50.99 | 53.09 | 57.43 |
| Backness | 64.48 | 63.72 | 66.39 | Backness | 52.32 | 52.59 | 55.52 |
| Roundedness | 54.23 | 74.52 | N/A | Roundedness | 52.77 | 53.73 | N/A |
| 135 ms |  |  |  | **135 ms** |  |  |  |
| Height | 84.35 | 83.54 | 84.23 | Height | 80.08 | 79.47 | 82.84 |
| Backness | 83.79 | 83.77 | 84.27 | Backness | 80.31 | 79.80 | 80.76 |
| Roundedness | 79.78 | 87.72 | N/A | Roundedness | 80.19 | 80.40 | N/A |

**Supplementary Table 2.** Participants’ response accuracy percentages based on articulatory movement conditions across the gates

## Supplementary Figures

**Supplementary Figure 1.** Center of gravity averages over time based on articulatory movements

**
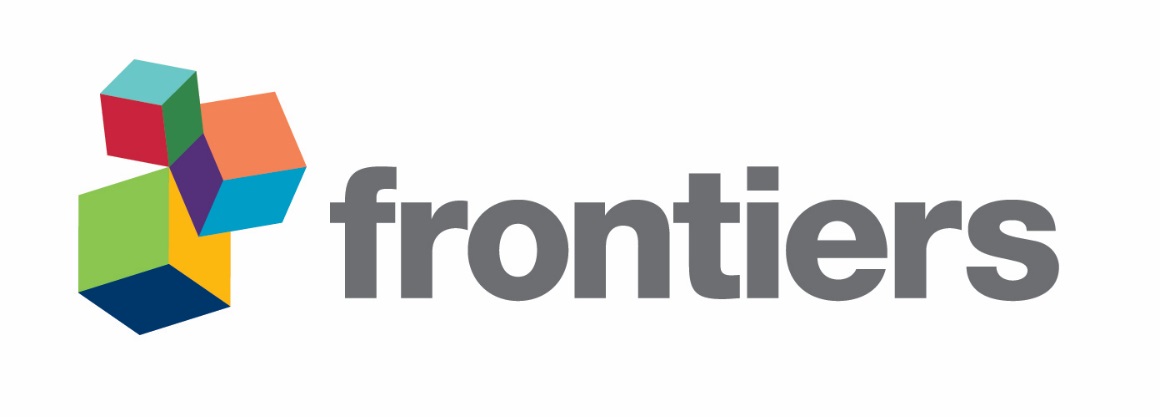
**
